# Children with a rare congenital genetic disorder: a systematic review of parent experiences

**DOI:** 10.1186/s13023-022-02525-0

**Published:** 2022-10-17

**Authors:** Charlotte von der Lippe, Ingrid Neteland, Kristin Billaud Feragen

**Affiliations:** Centre for Rare Disorders, Rikshospitalet, Oslo University Hospital, P.B. 4950, 0424 Nydalen, Oslo, Norway

**Keywords:** Rare genetic disorder, Child, Parent experiences, Qualitative, Systematic review

## Abstract

**Background:**

Caring for a child with a chronic disease may be demanding and stressful. When a child has a rare condition, the impact of care on parents is amplified due to the rarity of the diagnosis. In order to address the lack of generalized and synthesized knowledge regarding parents’ experiences of having a child with a rare genetic disorder, and give a holistic picture of these experiences, a systematic review of the available qualitative research was conducted.

**Methods:**

We performed a systematic review, including qualitative studies on parents of children with rare genetic disorders, published between 2000 and 2020.

**Results:**

The review included 33 qualitative studies. Findings were synthesized and categorized according to three main themes: Parents’ experiences with health care, Responsibilities and challenges, and Factors promoting positive experiences in parents. The findings demonstrate that parents of children with rare genetic disorders share many common challenges, despite evident differences across conditions.

**Conclusion:**

Coordinated care, and a more holistic approach in the follow up of children with rare genetic disorders is needed. International collaboration on research, diagnostics, producing scientific correct and understandable information available for health care professionals and lay people should be prioritized.

**Supplementary Information:**

The online version contains supplementary material available at 10.1186/s13023-022-02525-0.

## Introduction

Rare disorders are medical conditions that affect less than 1:2000 individuals or fewer [1]. In the USA, a disease is considered rare if it affects less than 200, 000 (~ 1:1600) individuals [2]. Most rare disorders are associated with a genetic cause [3].

Although rare disorders are rare by definition, it has been estimated that a rare disorder affects as many as one in 16 people [4]. Rare disorders are often chronic, with various degree of physical and psychological consequences [5, 6]***.*** Many rare disorders are congenital and identifiable at birth. For a few rare disorders, treatment may be available [7], however, for most there is only, if any, symptomatic treatment.

Caring for a child with a chronic disease may be demanding and stressful [8, 9], and caregivers of children with health problems have a greater risk of having health problems than those of healthy children [10]. When a child has a rare condition, care demands may be complicated and possibly amplified because of the rarity of the condition, and parents of a child with a rare diagnosis may therefore experience increased physical and emotional stress [11–13]. However, parents of children with chronic diseases may also experience positive aspects of parenting, such as increased personal strength and greater appreciation for life [14].

There are between 6000 and 8000 rare diseases, and it has been estimated that rare conditions may affect as many as 30 million Europeans and 25 million North Americans [15, 16]. Hence, many children and their families across the world have to live and cope with the medical, psychological, and social consequences of the rare condition. Due to a low prevalence of each rare disorder, knowledge about most rare disorders is sparse both in society and among health care professionals. Consequences of the lack of knowledge about rare disorders may lead to diagnostic mistakes, delays in diagnosis, and lack of information of high quality [17–19].

Increased awareness of rare disorders throughout society, and within the health care system, is one suggested action to improve the situation of people with rare disorders [20, 21]. With 6000–8000 different rare conditions, the understanding of common experiences that may be present across conditions can be difficult to assess. Therefore, one way to increase knowledge, is to summarize research investigating psychological and social experiences of parents of children with rare disorders across conditions. A synthesis of qualitative studies may benefit from the depth of understanding uncovered by each qualitative inquiry, while also identifying shared experiences identified across studies, and their consequences in everyday life, which may shed light on unmet needs that require coordinated societal responses.

Qualitative methodology [22] is ideally suited for investigating the psychological, emotional, and social specificities of being the parent of a child with a rare genetic disorder, in order to gain deeper insight into people’s experiences and seeking to understand the meaning or nature of these experiences. Nevertheless, there is a lack of qualitative research exploring parents’ experiences of having a child with a rare genetic disorder, and whether these parents face challenges that are qualitatively different from those experienced by parents of children with more well-known medical conditions. Further, few papers include several different diagnoses in the same study, so that similarities and differences across conditions can be investigated from a psychological perspective, and last, a lack of literature reviews summarize shared experiences of parents of children with a rare genetic disorder.

### Aims

In order to address the lack of generalized and synthesized knowledge regarding parents’ experiences of having a child with a rare genetic disorder, we conducted a systematic review of the available qualitative research on this population, in order to provide a holistic picture of common experiences across different diagnoses.

The aims of this systematic review were:

1. To provide an overview of parents’ experiences of having a child with a rare genetic disorder, and explore the psychosocial consequence of these experiences.
2. To address the overarching question: What experiences do parents of children with rare genetic disorders share?

## Materials and methods

### Inclusion and exclusion criteria

**Fig. 1:**
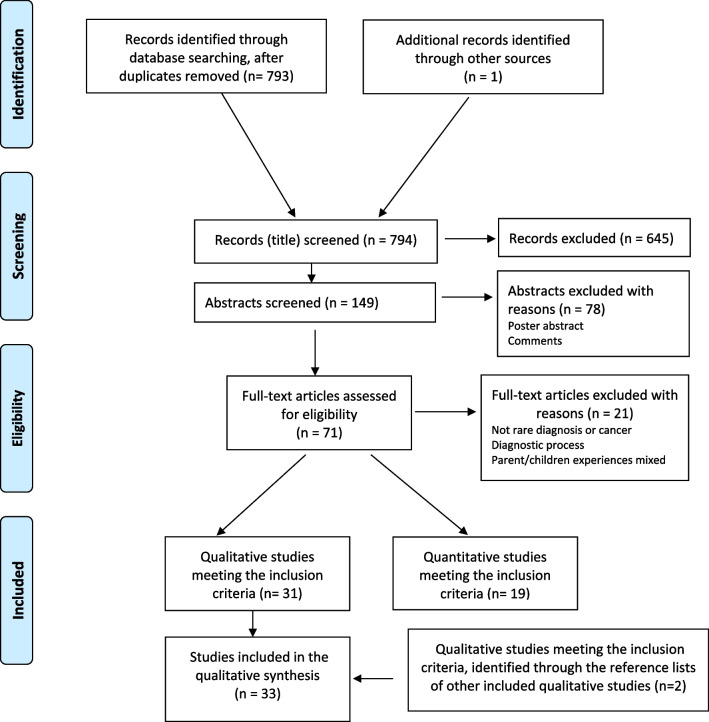
Flowchart of identified and selected articles

A systematic review of the qualitative literature was performed, following the PRISMA statement [23]. A flow chart of the number of identified and selected articles can be found in Fig. 1. All original, peer-reviewed articles published in English, addressing parents’ or primary caregivers’ experiences of having a child with a rare congenital genetic condition, based on qualitative or quantitative methodology, and published from January 2000 until November 2020 were included in the search. Quantitative articles were included in the search in order to get an overview also of the quantitative literature of the topic. The quantitative articles were not included in the qualitative synthesis.

Case studies were excluded. Studies on rare cancers, rare rheumatologic disorders, or rare acquired disorders were excluded, as many of these disorders do not have a clear genetic cause. Studies focusing mainly on the diagnostic process or with a focus on the use of internet were excluded. Reports, oral presentations or abstracts from posters were excluded.

### Search strategy

The PROSPERO International prospective register of systematic reviews was searched to be sure a similar study was not started, and a protocol for this study was published (Prospero CRD42018111129).

The search strategy was developed in cooperation with a specialist librarian. We searched the following electronic databases to identify relevant studies, number of hits in parentheses: Ovid Medline (668), APA PsycInfo [70], Web of Science (163). Date of search was December 3rd, 2020. Total number of hits was 901. Number of hits after removal of duplicates was 793. We used the search words: rare, orphan, diseases, disorder*, diagnosis*, condition, parents, fathers, mothers, single parent, single-parents family, maternal behavior, paternal behavior, parent–child relations, father-child relations, mother–child relations, parenting, child rearing, caregivers, professional family relations, family, family relations, family conflict, parent*, caregiver*, caregiving, carer, carers, mother, father, maternal*, paternal*,family*, families, experienc*, lived experienc*, cope*, coping, parental characteristics, parental attitudes, parental role, parenting skills, parenting style, childrearing practices, child discipline, parent child communication, parent child relations, childrearing attitudes, parental involvement, including MeSH terms.

The search was restricted to English language, key words, titles and abstracts, and publication time was restricted to January 2000–November 2020.

### Selection of included papers

Search results were merged using EndNoteX9 and duplicates were removed. Three independent reviewers examined the titles and abstracts, and selected papers for full-text reading. All three reviewers read full-text of selected papers, and papers were included in the study according to the agreed criteria (Additional file 1: Appendix I). Questions used to include or exclude publications after full-text reading were (1) Is the study empirical and in English? (2) Is the child’s diagnosis rare and genetic? (3) Is the study about experiences of being parent to a child (any age)? (4) Is the study qualitative or quantitative?, and (5) Does the study follow standards for reporting qualitative research [24].

If the answers to questions 1- 3, and 5, were yes, and the study was qualitative, we included the study in the synthesis. Any potential disagreements between the authors were resolved through discussion.

### Data extraction

All three co-authors collected data regarding citation/contact details, methods, design, participants, setting/context and results/findings (Additional file 2: Appendix II).

### Data synthesis

Qualitative research is specific to a particular context, time and group of participants, and caution is therefore needed when generalizing results. Having this in mind, it is however possible to extract results from different qualitative studies, and synthesize findings. Several methods for synthesizing qualitative data have been recommended [25], and thematic synthesis [26] was employed in the present review. All findings were extracted from the included studies’ result sections. Following extraction, the text was coded, and codes were grouped into meaningful categories, so called descriptive themes. CvdL and IN independently synthesized the data extracted, before discussing themes. Subsequently, KBF, familiar with all included papers, reviewed the themes before going through the codes to check whether they had been included in the themes. All three authors agreed on the final themes. The synthesis presents the overall findings in analytical themes and subthemes, and as presented by the authors in the publication’s result section. Rare genetic disorders are referred to as ‘rare disorders’ in the Results and Discussion.

## Results

In total, 33 qualitative articles were included, representing a wide range of rare diagnoses and conditions. An overview with details of the included articles can be found in Table 1.

**Table 1:** Overview and details of included studies

Reference	Country	Diagnoses	Sample size (#caregivers)	Age range of the children	Methodology
Baumbusch et al. [27]	Canada	Several	16	6 weeks–16 years	Thematic analysis
Brewer et al. [28]***	UK	Juvenile Huntington’s disease	12	7 teenagers 1 < 10 years 4 > 20 years	Interpretetive phenomenological analysis
Bruns & Foerster [58]*	USA	Trisomy 9, 13, and 18	20	40–370 months	Thematic analysis? (not specified)
Bruns & Schrey [59]*	USA	Trisomy 9, 13, 18	20	40–370 months	Thematic analysis? (not specified)
Cardinali et al. [29]	Italy	Several	15	N/A	Grounded theory
Currie & Szabo [30]**	Canada	Several	15	< / = 15 years	Interpretetive thematic analysis
Currie & Szabo [31]**	Canada	Several	15	< / = 11 years	Hermeneutical phenemenology
Currie & Szabo [49]**	Canada	Several	15	< / = 11 years	Hermeneutic phenomenology
Feragen et al. [32]	Norway	Congenital craniofacial anomaly (CFA)	48	One year–18 years	Inductive thematic analysis
Germeni et al. [50]	Italy	Bartter syndrome	13	N/A	Thematic analysis
Gerstein et al. [33]	United States	Urea cycle disorders	35	0—> 18	Thematic content analysis
Gilmore et al. [34]	Australia	Several chromosomal disorders	22	1–17 years	Thematic analysis
Glenn [35]	USA	Alagille	16	6 months–17 years	Hermeneutic phenomenology
Gómez-Zúñiga et al. [36]	Spain	Several	10	N/A ?	Grounded theory
Griffith et al. [37]	UK	Cri du Chat, Cornelia de Lange, Angelman syndrome	8	Adults	Thematic content analysis
Güeita-Rodriguez et al. [55]	Spain	Rett syndrome	31	Mean age 12.57 (SD ± 9.02) years	Inductive thematic analysis
Kleinendorst et al. [39]	Netherland	16p11.2 deletion syndrome	23	Median age 9 years (0–> 12 years)	Thematic analysis
Lim et al. [40]	China	Rett syndrome	14	4—18 years	Content analysis
Pousette Lundgren et al. [41]	Sweden	Amelogenesis imperfecta	8	N/A, but < 18 years	Thematic analysis
Myrin-Westesson et al. [38]	Sweden	Hemophilia	14	1–24 years	Hermeneutic phenomenology
Nag et al. [42]	Norway, Sweden and Denmark	Smith–Magenis syndrome	48	1½–50 years	Phenomenological approach
Purcell et al. [56]	USA	Neuroendocrinehyperplasia of infancy	12	N/A	Grounded theory
Ragusa et al. [54]	Italy	Prader-Willi syndrome	138	> 5 years	Narrative based
Smith et al. [43]***	UK	Juvenile Huntington’s disease	12	7 teenagers 1 < 10 years 4 > 20 years	Interpretetive phenomenological analysis
Somanadhan & Larkin, [44]	Ireland	MPS I, MPS II, MPS III, and MPS VI	8	6 months–22 years	Hermeneutic phenomenology
Tikkanen, Peterson & Parsloe [57]	USA, Italy, Holland, New Zealand, Australia, Canada, and Montenegro	Sturge-Weber syndrome	24	N/A	Interpretetive thematic analysis
Trulsson & Klingberg [53]	Sweden	Several	14	3–21 years	Grounded theory
Vitale [45]	USA	Prader-Willi syndrome	20 parents	2–17 years	Thematic analysis
von der Lippe et al. [51]	Norway	Hemohilia A and B	16	N/A	Inductive thematic analysis
Weng et al. [46]	Taiwan	Silver-Russel syndrome	15	N/A	Content analysis
Wu et al. [47]	Taiwan	Epidermolysis bullosa	10	N/A	Phenomenological approach
Yang et al. [52]	Taiwan	SMA I & II	19	7–12 years	Phenomenology (Giorgi)
Zelihić et al. [48]	Norway	Bardet-Biedl syndrome	5	0–16 years	Thematic content analysis

*N/A* not applicable

^*^Same material used in two studies

^**^Apparently same material used in three studies,

^***^Same material used in two studies

The findings demonstrate that parents shared a range of common experiences despite the uniqueness of their child’s condition. Three main themes were identified: (1) Parents’ experiences with health care, (2) Responsibilities and challenges, and (3) Factors promoting positive experiences in parents. All main themes included subthemes, which will be subsequently described. An overview of themes and subthemes in relation to all included studies can be found in Table 2.

**Table 2:** Themes and subthemes presented in the included studies

Theme	Parents’ experiences with health care			Responsibilities and challenges				Factors promoting positive experiences in parents		
Subtheme/ reference	Health care professio-nals’ lack of knowledge and experience with rare conditions	Lack of coordi-nated health care	The many unknowns	Socity’s lack of infor-mation and knowledge	Changes and adjust-ments in everyday life (work, parent-hood, social life)	Parents as coordinator, advocates and experts	Emotional reactions	Engaged and under-standing health care professionals	Benefits of social support	Protective factors and coping mechanism
Baumbusch et al. [27]	x	x	x		x	x	x		x	
Brewer et al. [28]	x		x	x	x	x	x		x	x
Bruns & Foerster [58]					x		x		x	
Bruns & Schrey [59]					x		x		x	x
Cardinali et al. [29]	x	x	x		x	x	x		x	
Currie & Szabo [30]	x	x	x	x	x	x	x	x		
Currie & Szabo [31]	x	x	x	x	x	x	x	x		
Currie & Szabo [49]	x	x	x	x	x	x	x			
Feragen et al. [32]	x		x		x	x	x			x
Germeni et al. [50]	x	x	x	x	x	x	x		x	x
Gerstein et al. [33]	x	x	x		x		x		x	x
Gilmore et al. [34]	x		x	x		x	x	x	x	
Glenn [35]	x			x		x	x	x	x	x
Gómez-Zúñiga et al. [36]	x	x	x	x	x	x	x	x	x	
Griffith et al. [37]	x	x	x	x	x	x	x	x		x
Güeita-Rodriguez et al. [55]	x	x		x	x	x	x		x	
Kleinendorst et al. [39]	x	x	x	x	x	x	x		x	
Lim et al. [40]	x	x	x			x	x		x	
Pousette Lundgren et al. [41]	x		x	x	x	x	x	x	x	x
Myrin-Westesson et al. [38]	x		x	x	x	x	x			x
Nag et al. [42]	x		x	x		x	x	x	x	x
Purcell et al. [56]			x		x		x		x	x
Ragusa et al. [54]	x		x		x		x	x	x	
Smith et al. [43]	x		x	x	x		x			
Somanadhan & Larkin [44]	x	x	x	x	x	x	x	x	x	x
Tikkanen, Peterson & Parsloe [57]				x	x				x	x
Trulsson & Klingberg [53]	x	x	x	x	x	x	x	x		x
Vitale [45]	x	x		x	x	x	x		x	x
von der Lippe et al. [51]	x				x	x	x	x		
Weng et al. [46]	x		x	x	x	x	x			
Wu et al. [47]	x		x	x	x	x	x		x	x
Yang et al. [52]	x	x	x	x	x	x	x		x	x
Zelihić et al. [48]	x	x	x	x	x	x	x	x	x	x

### Theme 1: Parents’ experiences with health care

All studies except three explored parents’ experiences with health care services in charge of their child’s follow-up. The first theme was further categorised into three subthemes: Health care professionals’ lack of knowledge and experience with rare conditions, Lack of coordinated health care, and The many unknowns in terms of prognosis, treatment, and function.

### Health care professionals’ lack of knowledge and experience with rare conditions

Twenty-nine of the papers raised issues related to an experienced lack of knowledge about and experience with rare conditions among health care professionals. As a consequence, parents experienced uncertainties regarding the child’s diagnosis, prognosis, treatment and/or consequences of the rare condition [27–49]. More specifically, parents reported diagnostic delays [27, 29, 50], and health care professionals that could not provide the information they needed about the rare condition once diagnosis was set [27, 30, 34–36, 39, 48]. Parents did not receive the guidance normally provided within the health care system [27, 33, 41–43], which could lead to a loss of trust and confidence in those who are meant to be the experts [32, 46, 51, 52]. Other consequences of a lack of knowledge within the health care system could be the unintended consequence of delaying treatment [27, 37]. Parents felt frustrated or troublesome when health care professionals did not understand what they believed to be their child’s health care needs [35, 41, 42].

The lack of knowledge within the general population strengthened the parents’ needs for health care professionals to have relevant, deep, and extensive knowledge and expertise [53]. One study specified that it was not the lack of competence or knowledge per se that parents found difficult, but what they perceived as the physicians’ attitude; their (un)willingness to admit their shortcomings and to seek information and advice [51] or to properly prepare before the consultation [30, 54].

Several categories of health care services were mentioned in the included studies, ranging from specialized health care services (such as specialized hospital settings and treatment teams), local health care services (such as general practitioners, local hospitals), and professional caregivers in the families’ homes (such as health care assistants). Eight studies specifically raised the issue of a lack of knowledge and diagnostic expertise within local levels, even after the child’s diagnosis had been set [31, 33, 37, 40, 49, 51, 52, 55].

### Lack of coordinated health care

Several studies mentioned a general lack of coordination across systems or sectors in the plan of care for the child with a rare condition, even in cases of complex care needs or long-term intensive support [27, 29, 31, 36, 49, 52]. Several studies included overwhelming parental narratives of fragmented care, with medical teams working in silos instead of integrating the family’s needs, leading to repeated consultations and numerous medical appointments with a range of clinicians in different hospitals [27, 29–31, 37, 40, 44, 50]. A lack of coordinated care could contribute to a delayed diagnosis [55] and feelings of depersonalization, since parents had to tell and re-tell their story to new health care providers [31].

Parents believed that treatment of rare conditions should be organized within standardized and specialized follow-up care systems or centers of expertise with a main health care provider to coordinate care [39, 44]. In cases where parents had received advice and follow-up from specialized units, this was experienced as positive and strengthened their trust in the quality of the child’s care [33, 45, 48]. Having the same caregivers over time was perceived as extremely important for families, because it led to enhanced availability and continuity [53]. In one study however, parents explicitly said they did not feel the condition’s rarity was an issue, and they therefore did not feel a need for specialized support services [37].

### The many unknowns

The lack of knowledge within the health care system led to many unknowns due to a delayed or complicated diagnostic and treatment process with several consultations [27, 30, 31, 38–41, 47–49, 53, 54]. The diagnostic process and first phase of the child’s life had therefore been demanding for many parents [32, 40, 48, 53, 56]. The longer and more complex the diagnostic process, the more stress the parents felt [50]. Although knowing their child had a rare condition was distressing, receiving a diagnosis was experienced as a relief and a first step towards treatment and support [40, 50]. Parents felt that they were responsible for the next steps after a diagnosis was set [27], but the complexity of the child’s diagnosis could complicate their understanding of what was to come [36].

The many unknowns triggered parents’ feeling of being abandoned to their fate, having to cope with the child’s illness on their own, and with an overall feeling of not being understood [34, 36], which complicated the parents’ process of adjustment and coping [28, 37, 50].

Caregivers had several questions regarding the child’s future, and were worried about whether their child would be capable of doing things independently, how cognitive development would unfold, and whether the child would be able to live on their own in the future [29, 33, 39, 44, 46, 52]. The many unknowns called for more support and guidance [39, 42, 43]. However, advices from health professionals could be inadequate and vary across levels of health care services [34, 42], and limited evidence-based guidance complicated parents’ efforts to understand and compare risks and benefits when considering treatment alternatives [33].

### Theme 2: Responsibilities and challenges

All studies described how parents experienced responsibility for their child’s medical care and handled challenges associated with the child’s diagnosis and everyday life. Theme 2 was categorised into four subthemes: Society’s lack of information and knowledge, Changes and adjustments in everyday life (work, parenthood, social life), Parents as coordinators, advocates, and experts, and Emotional reactions.

### Society’s lack of information and knowledge

Parents often spent considerable time explaining their child's condition when meeting new people in settings such as playgrounds, shopping centres, or schools, an information task some parents experienced as demanding [28, 45, 48, 49, 53, 57]. Nevertheless, they felt responsible for raising awareness about the rare condition [45, 57], even when it felt difficult to explain to other people what their daily life looked like [31, 34, 35]. The challenge of explaining could be even greater if the child’s diagnosis was not visible to others, since caregivers could struggle to explain the child’s needs for special support [39, 43]. The condition’s complexity could complicate the process of sharing information to others, especially if parents did not feel knowledgeable themselves to adequately explain [50], and parents missed reliable sources of knowledge where they could find information [30, 31, 36]. Lack of knowledge also had consequences in school settings [28, 39, 42] or public institutions when applying for social rights or benefits [55]. In some studies, the lack of understanding was a challenge also within the extended family, which reduced the possibilities of social support [45, 47, 52].

Social experiences among strangers and a general lack of knowledge in society could be demanding due to staring or comments if the child looked different or behaved differently [28, 37, 38, 41, 43, 44, 46, 49, 50, 57]. Questions from others and/or a need to explain the difference was experienced as demanding by some parents [45], and some used preemptive and active strategies, hoping to fend off questions and stares [57]. Parents also described anticipated or experienced social stigma and taboo as challenging [44, 46, 49, 50, 57].

### Changes and adjustments in everyday life (work, parenthood, social life)

Parents described how having a child with a rare condition had an impact on the whole family, siblings included [29, 32, 33, 38, 41, 44, 45, 48, 50, 52, 53, 55, 58, 59].

Responsibility for the children and their care was described as intensive and demanding, and affected parents’ day-to-day living [37, 39, 46, 49–51]. Coping with challenging day-to-day experiences and in some cases living in high alert over time was described as exhausting [31, 36, 49]. Because of the many daily challenges, levels of conflict could arise between spouses/partners and affect their relationship [29, 32, 38, 44, 47, 52]. In contrast, three studies mentioned that the challenges could strengthen feelings of togetherness between the parents or within the family [29, 52, 58]. In one study, parents had specific recommendations for couples in order to preserve marriage and other relationships [45]. Lack of support in the larger family system could also lead to a higher level of conflict within the affected family [52]. Nevertheless, the priority was given to the child’s needs [29, 45, 58].

Some rare conditions present with specific behavioural or medical challenges with an impact on the family’s daily life. Hence, parents had to handle nutritional problems [53], food-seeking behaviours [39, 45, 54], communication problems [39, 43, 53], and behavioural problems [49]. The child’s condition could affect, complicate, or challenge the parent–child relationship, due to problems with communication and cognitive functioning, and/or behavioural characteristics that could be associated with the condition [28, 43, 45, 48, 53]. Treatment demands could break the child’s trust in the parents as their guardians against painful experiences [32, 38], also affecting the parent–child relationship. Difficulties were especially challenging when the child could not express his or her own needs, making it very difficult for the parents to know whether their child was in pain or was in need of something [48]. During adolescence and early adulthood, parents mentioned how adherence issues to treatment could reduce the child’s long-term independence, and rise concerns about their child's ability to manage their own medical needs [33], possibly also affecting the child-parent relationship. In social settings, parents felt the need to shield their child from other people’s attitudes, fearing that the child’s self-perceptions could be negatively affected if people reacted to the child’s behaviour or the rare disease [50, 57].

Demands associated with the rare condition led parents to feel torn between caring for their child and work obligations [27, 47, 49, 55]. They felt that they had to inform the work place about their situation [57] or seek a different work situation [29, 45, 54, 59], when the child’s care was described as a part-time job in itself [27, 59]. Additional care needs also led parents to struggle with finding time for personal and/or social activities [30, 46, 48, 55], and complicated the preservation of social relations outside the family [32, 39, 54, 56]. Plans were difficult to make or had to be adjusted to the situation because of the many insecurities associated with daily care and/or treatment demands [41, 51, 55].

Due to medical or psychological problems related to the child’s diagnosis, caregivers experienced difficulties in looking after their child and provide the best upbringing [39]. Hence, in-home caregivers were necessary in some families. Still, finding suitable in-home caregivers that parents felt they could trust, and welcoming them into their private home could feel challenging and invading [31, 59].

### Parents as coordinators, advocates, and experts

Due to the lack of knowledge within the health care system, parents were the ones finding out whether support was existing and available, requesting care, social aid or benefits, or other resources they in some cases did not manage to receive, and took on the arduous and demanding responsibility of coordinating the follow-up of their child [27, 29–32, 37, 39, 41, 42, 46, 48, 49, 55].

Several studies shed light on parents’ struggle to get what they believed should be proper care, being the ones noticing or bringing up that something was wrong with their child, being perceived as difficult and demanding, or having the feeling that health care providers did not believe them or even blamed them for the child’s symptoms [27, 29–32, 35, 37, 38, 40–42, 46, 47, 49, 51, 53]. Being dependent upon referrals and access to other necessary aids created a feeling of disempowerment in some parents, if such help was not provided [27]. Caregivers also felt they took on the responsibility for medical care they did not have any competence for in the first place, such as handling nutritional adjustments, educational needs, and/or managing other problems related to the diagnosis [47, 50].

Due to a lack of dialogue between health care professionals, parents experienced medical appointments as repetitive in nature, and the need to tell their child's and family's story repeatedly across consultations [30, 53]. Parents described spending energy and time looking for medical treatment that could alleviate their child’s symptoms [47], hoping to regain some control by taking on the responsibility of researching their child’s health care needs [32]. Some parents, or the larger family, also took the responsibility of finding and trying out treatment alternatives, in the hope of alleviating their child’s suffering [46, 52].

Parents also felt responsible for special arrangements in school, social activities, interpersonal relationships, general life adjustments and assistance from psychological support teams, in addition to the family’s financial security [28, 42, 44–47, 52]. One study described how school and health care settings also relied on parents’ knowledge and information to coordinate the child’s needs [42].

Parents labelled themselves as fighters, saviours, and navigators for their child, in their efforts to be heard [31, 37, 53] and described the paramount need to stand up for the child, intervene, negotiate, or act on the child's behalf, which could sometimes mean less time for caring for the sick child [44, 45].

The lack of knowledge about the child’s rare condition led parents to search for information on the internet, but missed guidance from health care providers on this search [27, 34, 35, 44]. They tried to be critical of the information they found and looked for what they considered to be reputable sources, such as scientific journals, and also connected with health care providers with specialized knowledge [27]. As a consequence of this extensive and ongoing search for information, in addition to their lived experiences, parents became experts on their child’s rare condition and felt they had acquired more knowledge about the rare condition than the health care providers [29, 30, 35–37, 51, 52, 55]. Parents could feel that care providers’ knowledge was based on outdated information, whereas they had read more recent studies and were more updated on relevant research [30]. Nevertheless, several studies revealed that some parents did not feel that their experience was valued, acknowledged, or sought by health care providers [31, 35, 36, 53]. This reversal of traditional parent–professional roles was experienced as difficult and an additional responsibility for some parents [28], who frequently felt they needed to be the expert “home doctors” [28, 35, 45, 50, 51]. Other studies showed that some parents treasured feeling as experts in their child’s care, and that understanding complex medical information could increase parents’ self-confidence [29, 32, 35].

Caregivers described how they had to monitor whether or not symptoms were developing in their child, for example whether their child was gaining weight or whether problems were related to the diagnosis or the child’s personality development [39], and in some cases also felt they were responsible for treatment decisions [28].

### Emotional reactions

Parents described a wide range of emotional reactions, such as feelings of shock, anxiety and fear, lack of control, defencelessness, depression or loss, denial, self-blame and guilt, helplessness, and distress [28, 29, 32–36, 38, 41, 42, 44–47, 49, 51–53, 55]. Uncertainty, unpredictability, and ambiguity characterized everyday life for many parents, or they felt trapped in a box or square that they could not get out of [43, 44, 46, 53]. Feelings such as disbelief, displacement, anger, frustration, or pain were also described [29, 31, 35, 37, 40, 44, 45, 54], eventually followed by feelings of acceptance [47, 53]. In cases of genetically inheritable disorders, parents also felt guilt or fear of passing on the disorder to their children [36, 41]. Life was described as a rollercoaster or a constant battle [37, 38, 44]. In one study, parents described how they felt they were in a movie, watching something they struggled with understanding was their own life, being centre stage and managing complications and disease manifestations, they had never imagined [49]. Having to cope with their child’s pain, fear of death, or the child’s own grief over the rare condition acted as an additional worry for parents [38, 43, 44, 52, 53].

Parents suffer because, firstly, their child’s illness requires so much attention, time, and energy that the physical and emotional wear and tear sooner or later takes its toll [36]. The many emotional reactions, such as powerlessness, threatened the parents’ belief in their own parenting skills [32, 38]. Several studies also shed light on physical symptoms of exhaustion, physical burnout, insomnia, or illness in parents of children with a rare condition [30, 32, 36, 42, 47, 59].

The concern regarding potential social reactions was a reality for many parents [28, 43, 46, 47]. Informing others was associated with feelings of depression and anxiety [48]. Fear or experiences of bullying was also a prominent aspect for several parents [41, 46]. Parents also described the immense emotional cost of shielding or defending their child against social misconceptions and reactions, due to the social or physical visibility of the condition, sometimes leading to social avoidance [28, 32, 37, 38, 41, 43, 44, 49].

Having to cope with many unanswered questions regarding the child’s future care and treatment options caused feelings of loneliness, helplessness and insecurity [27–29, 32, 34, 36–40, 44, 47, 49, 52, 54–56]. Fragmented care delivery increased families’ emotional load [30, 37, 44, 50]. The overall lack of understanding and knowledge about the rare genetic disorder and its treatment led to anger, frustration, sorrow, and feelings of isolation [28, 42, 44], or a sense of loneliness [36, 50], due to the lack of strategies or tools needed to deal with the situation. Parents could find it difficult to share their experiences and what they went through, which led to feelings of isolation [31, 34, 48, 49]. Feeling isolated could also be triggered by a lack of understanding from close friends or family [49, 58], or from health care providers [27, 29, 30, 41, 52]. In contrast, social support and normalising everyday life, such as going to work, reduced feelings of isolation [48, 56]. Parents were also concerned over how the impact of illness affected their child's quality of life and/or daily life [28, 33, 43].

### Theme 3: Factors promoting positive experiences in parents

All studies except two presented findings related to positive adjustment in parents of children with a rare condition. The third theme was categorised into three sub-themes: Engaged and understanding health care professionals, Benefits of social support, and Protective factors and coping mechanisms.

### Engaged and understanding health care professionals

Parents shared how relieving it was to be treated with respect and knowledge from the health care professionals in charge of treatment and feel that their problems were taken seriously [41]. Care professionals honouring the families' knowledge and recognising that parents had first-hand experience with the condition was important [30, 31]. The development of self-reliance and trust in their ability to cope with problems could be enhanced when parents’ perception of subjective vulnerability was counterbalanced by support from professionals [35, 53].

The importance of professional caregivers’ personal characteristics was underlined, so that a trusting relationship could be built between parents and helpers [53]. Respect, compassion and empathy, emotional support and involvement, being treated with sensitivity, tact, and kindness, continuity, knowledge and availability, and boosting parents’ knowledge were described to be ideal characteristics in health care professionals [34, 36, 48, 53, 54]. Personal and direct communication was also central when information was provided [44]. Connection with care professionals was achieved when they were experienced to be kind, caring, present, understanding and listening, while also being real and truthful about the situation [30, 37, 42, 48, 53, 54]. Trust depended on the degree to which professionals managed to be honest about their lack of knowledge and managed to show that they understood the emotional impact of the rare condition on the families’ lives [36, 48, 51].

### Benefits of social support

Social support was experienced as hugely important, protected against emotional distress [35, 48, 56, 58], and provided parents with much necessary support when the child’s help needs exceeded the parents’ available resources [36]. Daily life, such as being at work, normalised parents’ situation and enabled them to have social interactions, which could have a protective social function [48, 54, 59]. Social and emotional support could also be found in faith communities and helped parents coping with their situation [56]. Specific and practical support, on the other hand, was complicated by parents’ fear that others could not correctly understand their child’s care needs and they therefore could not trust support to be given [45]. In one study, fathers did not want social support, since handling things alone or within the nuclear family acted as a protective strategy and a buffer against exposure to the courtesy stigma that could be triggered if help was sought or received [57].

The larger family may normally provide additional support, which was confirmed in one study [58]. However, cultural or societal frameworks could lead the larger family, such as older family members and grandparents, to blame the child’s parents for the rare condition [47, 52], or feel shame about their grandchildren, which led to a lack of support within the larger family [52]. In yet other families, the genetic aspects of the condition meant that several family members were affected; reducing the opportunities for support, and/or caregivers could find it difficult to ask for help [28].

Other people’s level of understanding and positive attitude was described as central for parents to feel supported by friends and others [45]. Therefore, the emotional, practical, and social benefits of talking to others with similar experiences was highlighted as important by parents in several studies [27–29, 35, 36, 39–41, 45, 47, 48, 50]. Being active members of patient associations where parents could discuss challenges, share experiences, and provide each other with information and advice, was described as a main source of social support [27, 29, 44, 45, 50], and a necessary asset for reducing feelings of isolation [27, 29, 33, 35, 39, 48, 50]. Nevertheless, some parents felt that attending support conferences and meeting other parents had increased their worries for the child’s future [45].

The lack of knowledge within the health care system and society as a whole, leading to an absence of clear, understandable and accessible public information, strengthened the importance of searching for information on the Internet and seek support and feel connected to other parents who had undergone the same situation [27, 34, 35, 40, 42, 45, 55]. The asset of online peer support was described to be its flexibility and availability, with easy access to other parents’ experiences and recommendations on a daily basis or whenever needed [27, 35, 44]. Parents were, however, well aware that the Internet also could be an anxiety provoking and frightening tool [35, 44].

### Protective factors and coping mechanisms

Several studies mentioned individual characteristics that had strengthened parents’ coping mechanisms. Willpower, perseverance, and courage seemed particularly important, as well as the ability to adjust and plan everyday life so that it matched the child’s needs [35, 37, 42, 45, 47, 48, 52]. A sense of agency and self-reliance also strengthened parents’ ability to cope and trust in their ability to help and care for their child when problems arised [32, 33, 35, 37, 38, 53, 57]. High levels of health literacy was also explicitly described as helpful in one study [35]. Parents also aimed at increasing their child’s sense of agency, encouraging the child in participating in treatment decisions or defending him-/herself from negative social reactions [52, 57]. Parents had also experienced that demanding experiences had strengthened their self-confidence, changed their outlook on life, and increased their empathy skills and understanding of other’s challenges [32].

Families described a process of normality reconstruction, incorporating the child’s condition with its consequences, and a re-organizing of family life based on the needs of the child, which appeared to give parents a sense of control over their situation [50]. Having the same condition as their child was also described as enhancing parents’ coping skills, as they had previous experience with the disease [41]. Normalization and acceptance was facilitated if the parents felt the child’s situation was stable. Nevertheless, the lack of knowledge regarding the condition’s progress and outcome created a fragile sense of control, and could be easily shattered in case of unexpected events [44, 50].

Parents developed strategies and knowledge themselves, learning by doing [42]. Focusing on daily tasks and everyday life was a way of coping with grief and loss [28, 52]. Religious beliefs, or mindfulness practice and yoga, were described as helping caregivers revisit life's challenges, accept trials and tribulations, and find strength to cope [45, 47, 52, 56]. Parents described the importance of identifying activities or daily routines that could strengthen their own and the family’s emotional coping [45, 59]. The importance of focusing on positive aspects of being a parent of a child with a rare condition [44], as well as feelings of gratitude and hope also strengthened parents’ adjustment to the rare condition [38].

## Discussion

Parents’ experiences of having a child with a rare genetic disorder have previously not been systematically reviewed. The present review examined the qualitative literature methodically, in order to identify parents’ experiences of having a child with a rare genetic disorder. Findings were categorized according to three main themes: Parents’ experiences with health care, Responsibilities and challenges, and Factors promoting positive experiences in parents. This systematic review demonstrates that parents of children with rare genetic disorders share many common challenges, such as a lack of knowledge in the health care system as well as in society in general, a lack of coordinated care, and lack of available information about rare disorders. Consequently, parents experience that they have to be experts on their child’s rare disorder, coordinators in the health care system, and act as advocates for their child. Many parents felt isolated and alone, and experienced a change in their social situation when they became parents to a child with a rare disorder; especially mothers described challenges with working fulltime and having a child with a rare disorder. Few articles focused primarily on protective factors or parents’ coping mechanisms. However, the synthesis of the results demonstrated that all but two studies presented findings that shed light on factors promoting positive experiences in parents, such as engaged and understanding health care professionals, benefits of contact with others in a similar situation and social contacts in general, and the use of personal coping mechanisms such as educating themselves, focusing on daily activities, religious beliefs and feelings of gratitude and hope.

### Parents’ experiences with the health care system

Parents mentioned health care professionals’ lack of knowledge and lack of experience about rare disorders in the majority of the studies. Lack of knowledge, and its negative consequences such as delays in obtaining an accurate diagnosis and maltreatment [60, 61], is not novel news. Lack of knowledge is indeed a major barrier for people with rare disorders [62], and our systematic review demonstrates that this also is true for parents to children with rare disorders.

In 2009, the European commission requested that all European countries should elaborate and adopt plans and national strategies for rare diseases. Sadly, this seems to be easier said than done [63]. Collecting and sharing knowledge across different countries, and for different rare disorders, are important methods to increase knowledge. Unfortunately, the small number of available individuals to include in the research on rare disorders adds an extra challenge to this task. The readers of published literature may also be few, giving this research low prestige and more difficult to fund [64]. International collaboration is therefore of major importance, and research programs for rare disorders across countries, such as projects promoted by the European Joint Programme on Rare Diseases (EJP RD) [65], should be encouraged. European Reference Networks (ERN) were founded on the principle that experts and specialists need to communicate and collaborate across countries if we are to solve challenges related to rare conditions [66]. However, the effect these ERN’s have on individuals’, families’ and health care professionals’ experiences on access to knowledge and treatment of rare disorders remains unanswered and should be prioritized in future research.

Some individuals live with an undiagnosed condition and the International Rare Diseases Research Consortium (IRDiRC) suggest that this group of individuals should enter a globally coordinated diagnostic and research pipeline [67]. Until such a pipeline is up and running, existing international collaboration is of immeasurable value. The importance of national and international networks, and databases such as DatabasE of genomiC varIation and Phenotype in Humans using Ensembl Resources (DECIPHER) [68] and GeneMatcher [69], to identify other ultra-rare patients and researchers interested in the gene or disease cannot be overestimated.

Health care professionals and patient support organizations must continue to work together as they already do in the North American National Organization for rare disorders (NORD) and European Organization for rare diseases (EURORDIS). Although the awareness about rare disorders is increasing in Asia [70], there is room for improvement, especially in Africa [71]. Results from the current study demonstrate clear unmet medical needs, lack of knowledge on a societal level, with corresponding psychological consequences for parents of children with a rare disorder, problems that may be exacerbated in countries with less available resources. Hence, European and North-American actions, such as the organization of ERNs or NORD, could possibly have the potential to address some of the unmet needs revealed in the present study, and inspire similar actions in regions with fewer resources world-wide.

Several of the studies mentioned that the children had to see several different specialists before the diagnosis was set. Challenges continued also after the diagnosis, since far from all questions parents had had been resolved. For rare disorders, and especially for ultra-rare disorders, the current study confirms that parents face many unknowns, just to mention a few: What is the prognosis? Will there be treatment available? Will my child get access to treatment? For more well-known chronic disorders, parents will not need to ask most of these questions, because answers are obvious and health care professionals may provide them immediately. In contrast, parents of children with rare disorders often continue to search for knowledge about the disorder and possible treatment. Lack of coordinated care was identified as a major challenge for the parents in the present review. When parents of children with spinal muscular atrophy were asked to provide advice that could improve the follow-up of their child, they suggested health care professionals to designate a coordinator for every family [72]. Future research should investigate whether this is a solution that could improve parents’ health care experiences when the child has a rare condition.

### Responsibilities and challenges

In addition to health care professionals’ lack of knowledge, many parents described a lack of available information about their child’s rare disorder, and a general lack of knowledge in society. The parents described how they became the experts on the rare disorder, acted as coordinators for their children’s follow-up, and became advocates for their child. A review on adults with a rare disorder also revealed that people affected by a rare condition considered themselves as “expert patients”; They educated themselves and became experts on their condition, because of health care professionals’ lack of knowledge and experience with the condition [62]. Health care professionals should see this gained expertise as a value [73]. However, research may indicate that some health care providers feel challenged by lay knowledge [74]. Instead, health care professionals should use the expert knowledge parents of children with rare disorders have as a valued resource that may optimize care. Previous research has shown that the parents’ voices are vital to influence and guide service development [75], and a critical element in creating responsive, meaningful, and widely accepted policies [76].

Several studies demonstrated how care needs and consequences of the rare disorder had forced parents to make changes in their social life, such as cutting down work-hours or quitting their job, and seeing friends and family less. For some, this had promoted a sense of isolation and almost all studies described how parents had to cope with a range of emotional reactions in their daily lives that could potentially affect their psychological adjustment. Parents of children with a rare condition have additional stressors, including balancing work and family, time constraints, stress, and feelings of “doing it all” [77]. Research on rare craniofacial conditions has demonstrated that parental distress has the potential to impact the child’s own emotional development [78]. In contrast, parents who feel they have managed to adjust positively to their child’s condition will probably be better equipped to help their child to develop a positive and strong self-image [79], in line with research showing that parents’ sense of self-efficacy in their ability to care for their child is central for the development of the child’s well-being [80].

Although parents of children with congenital genetic disorders may have heritable concerns regarding their child’s genetic status, this was not a prominent theme in the studies included in this review. One reason may be that the issue of heritability was not specifically addressed in these studies. Concerns regarding heritability may be sensitive for parents to share, and thus may be missed unless specifically addressed.

### Factors promoting positive experiences in parents

Studies focusing primarily on factors and coping mechanisms that have a positive effect on parents of children with rare disorders are lacking. None of the included studies systematically investigated protective factors that could promote coping. Nevertheless, most studies revealed positive factors and parental coping mechanisms. As many of the negative factors, such as lack of knowledge and lack of treatment, may not be solved immediately, a focus on factors promoting positive experiences may be clinically helpful. Interestingly, in all but two of the studies, parents mentioned factors important to them as positive. Parents described the importance of having a social network and to be able to work outside of home in order to get some normalcy in life. It may therefore be important to encourage parents to continue in their jobs, and for society and employers to facilitate the work situation in an optimal way for parents [81], as well as encourage the parents to find ways to keep up their social life and contact with family and friends.

The parents considered it very beneficial to be in contact with others in a similar situation, i.e. parents of other children with the same diagnosis as their own child. For some rare disorders, there may be national or international patient support groups. For most rare disorders, this is missing, and parents may find support groups in social media such as Facebook. Information shared on support groups on Internet may be valuable to families with a member with a rare disorder [82]. A recent study demonstrated that most of the support groups on Facebook are private groups [83]. For many parents, these groups are the only place where they find others in a similar situation, as well as information about the disease and possible treatment options. A lack of professional involvement in these private groups may challenge the scientific quality of its content. Researchers and health care professionals could be more involved in such groups, as it could be of benefit to both parties. However, Facebook, or other similar web-sites on the Internet, are not secure platforms to share sensitive data, and parents and health care professionals should therefore be careful with their use.

An engaged and understanding doctor was also of high value to the parents, and these qualities in a health care professional seemed to be more important than the health care professional’s actual level of knowledge. Although health care workers’ lack of knowledge may be frustrating to parents, a lack of interest or a lack of respect for the parents’ knowledge may be even more damaging, and lead to a deterioration of the relationship between parents and health care professionals, which could be followed by less optimal health care for the child as a consequence. Though the lack of knowledge is disturbing, it is important to know that for some disorders, such as for example many ultra-rare disorders or disorders of *N*-of-1, little knowledge is available, and will perhaps be lacking for many years. It is therefore very important for health care professionals to show engagement, sensitivity, and understanding irrespective of the level of knowledge about the rare condition [72, 84]. Research on how health care professionals can provide optimal care for parents of children with rare disorders, despite a lack of competence and knowledge, should be prioritized, as well as research to minimize the gap of lack of knowledge. Health care professionals should be trained to handle situations where they do not have the necessary knowledge, and where information may be replaced by uncertainties. Meeting the parents with confidence, interest and respect will not act as a substitute to a lack of knowledge; however, it may still be of help to the parents. Less use of the health care system and poorer health may be the result of parents’ mistrust to health care professionals [85].

The majority of participants in the included studies were mothers. This could reflect that mothers take more responsibility for being the child’s primary caregiver. Indeed, several studies demonstrated that the father was the primary caretaker and provider of the family’s economy, by keeping a full time job. However, more research on fathers’ experiences is warranted.

### Strengths and limitations

The strengths of this literature review lie in the methodological and systematic approach, investigating the lived experiences of being a parent or primary caregiver of a child with a rare genetic disorder from a qualitative perspective. It is, however, also important to acknowledge some limitations with the present review. First, some methodological challenges were encountered. Given the many thousands different rare conditions, identifying a good search strategy was important, and the search strategy was therefore discussed in detail with a specialist librarian before conducting the search. A different methodological approach could have been to specifically include some more “common” rare congenital genetic disorders within the search process. However, choosing which diagnoses to include would have been a methodological challenge, and this method was therefore not chosen in the present review.

Articles on specific rare diagnoses in which “rare disease”, or its synonyms, were not included in the title, abstract or keywords, could therefore have been missed. Hence, chances were possibly higher not identifying rare conditions with higher prevalence rates, compared to very rare or ultra-rare conditions, and may have influenced results. However, in order to counterbalance this limitation, we used the search words rare, orphan, diseases, disorder*, diagnosis*.

Another methodological challenge was that some studies presented quotes without the context they were a part of, or presented some results very shortly, complicating the synthesis of the results in the present review. One paper presented their results as part of the discussion, also complicating the extraction of data for this review. Further, few studies explicitly explored the potential uniqueness of the rarity of a condition, investigating whether challenges that are identified have a similar or differential impact on individuals, depending of the specificity of the condition.

A strength of this study is that we are three authors with different backgrounds. CVDL and KBF both have experience in qualitative research. CVDL is a clinical geneticist with several years of experience of working with families with rare disorders. KBF is a psychologist and has vast knowledge about rare disorders and the psychosocial consequences of living with a rare disorder. IN is a doctor in training in pediatrics with less knowledge about rare disorders, which was seen as a strength, since IN could challenge CVDL and KBF’s potential pre-conceptions about rare disorders when discussing the synthesis of the results, reducing the risk of bias.

## Conclusion

The current review demonstrates that parents of children with a rare genetic disorder face many common challenges across different conditions. Health care professionals’ lack of knowledge seems to be a major obstacle for parent’s ability to care for their child, and they should be trained to handle and optimize meetings with the families in spite of uncertainties and lack knowledge. Parents also described the importance of having social networks and the benefit of being in contact with parents of children with similar challenges as themselves, which could possibly counteract the negative impact of a lack of knowledge in health care services and society in general. There is a need for more coordinated care for children with rare disorders, and a more holistic approach in the follow up of the children and the parents. The expertise of the parents should be valued. The development of more international collaboration on research, diagnostics, creating and making available scientific correct information understandable for health care professionals and lay people should be prioritized. Unmet medical needs and the lack of knowledge have clear psychological consequences for the parents, and therefore need to be addressed by health care policies.

## Supplementary Information

**Additional file 1**. Appendix I.

**Additional file 2**. Appendix II.

## Data Availability

All data generated or analyzed during this study are included in this published article.
