# Supplementary material for: Children with a rare congenital genetic disorder: a systematic review of parent experiences
Source: Orphanet J Rare Dis. 2022 Oct 17;17:375. doi: 10.1186/s13023-022-02525-0 (PMC9575260; doi:10.1186/s13023-022-02525-0)
Supplement: Supplementary file 1 — Additional file 1. Appendix I. [file 13023_2022_2525_MOESM1_ESM.doc]

**Appendix I: Questions to include or exclude publications after full text reading**

**Title, first author, year**:_____________________________________________­­­­­­­­­­­­­­­­_______

**1: Is the study empirical and published in full text?**

| ⁭ YES | ⁭ NO | ⁭ UNCLEAR |
| --- | --- | --- |

**2: Is the child’s diagnosis rare and genetic?**

| ⁭ YES | ⁭ NO | ⁭ UNCLEAR |
| --- | --- | --- |

**3: Is the study about experiences of being parent/caregiver to a child (any age)?**

| ⁭ YES | ⁭ NO | ⁭ UNCLEAR |
| --- | --- | --- |

**4: Is the research design one (or more) of the following:**

| Qualitative methods (interviews, focus groups, textual analyses) | ⁭ YES | ⁭ NO | ⁭ UNCLEAR |
| --- | --- | --- | --- |
| Quantitative methods (quality of life and other types of surveys) | ⁭ YES | ⁭ NO | ⁭ UNCLEAR |

**5: Does the study follow Standards for reporting qualitative research (Ref. O´Brien et al, 2014):**

| ⁭ YES | ⁭ NO | ⁭ UNCLEAR |
| --- | --- | --- |

**Decision:**

| Include | ⁭ (All questions are answered with ”YES”) |
| --- | --- |
| Discuss | ⁭ (Some questions are answered with ”UNCLEAR”) |
| Exclude | ⁭ (Some questions are answered with ”NO”) |

**Final decision:**

| Include | ⁭ (All questions are answered with ”YES”) |
| --- | --- |
| Exclude | ⁭ (Some questions are answered with ”NO”) Question number: |
