# Supplementary material for: Children with a rare congenital genetic disorder: a systematic review of parent experiences
Source: Orphanet J Rare Dis. 2022 Oct 17;17:375. doi: 10.1186/s13023-022-02525-0 (PMC9575260; doi:10.1186/s13023-022-02525-0)
Supplement: Supplementary file 2 — Additional file 2. Appendix II. [file 13023_2022_2525_MOESM2_ESM.doc]

**Appendix II: Data extraction instrument for studies included**

| **Reviewer:__________________________________** | **Date:________________________** |
| --- | --- |
| **First author:________________________________** |  |
| **Journal:____________________________________** |  |

**Year published _______________________**

**Study Description:**

**Aims, scopes and/or themes:**

**______________________________________________________________________________________________________________________________________________________**

**Methods:**

**______________________________________________________________________________________________________________________________________________________**

**Participants:**

**______________________________________________________________________________________________________________________________________________________**

**National background:**

**___________________________________________________________________________**

**Findings:**

**______________________________________________________________________________________________________________________________________________________**

**___________________________________________________________________________**

**___________________________________________________________________________**

**___________________________________________________________________________**

**___________________________________________________________________________**

**___________________________________________________________________________**

**___________________________________________________________________________**

**___________________________________________________________________________**

**___________________________________________________________________________**

**___________________________________________________________________________**

**Conclusion:**

**______________________________________________________________________________________________________________________________________________________**

**Reviewer’s comments:**

**_________________________________________________________________________________________________________________________________________________________________________________________________________________________________**
